# Salivary and Serum Glucose Levels in Diabetes Mellitus Patients versus Control – A Randomised Control Trial

**DOI:** 10.25122/jml-2020-0062

**Published:** 2020

**Authors:** Avanindra Kumar, Tanoj Kumar, Manish Bhargava, Rachna Raj, Vikas Vaibhav, Jay Kishore

**Affiliations:** 1. Department Of Oral Pathology and Microbiology, Patna Dental College And Hospital, Patna, India; 2. Department Of Oral Pathology, Patna Dental College And Hospital, Patna, India; 3. Department Of Oral Pathology, Manav Rachna Dental College, Faridabad, Hayana, India; 4. Department Of Public Health Dentistry, Patna Dental College And Hospital, Patna, India; 5. Department Of Dentistry, Vardhman Institute Of Medical Science, Pavapuri, Nalanda, Bihar, India; 6. Bihar Government, Primary Health Centre, Sahdei Buzurg, Vaishali, Bihar, India

**Keywords:** Diabetes mellitus, glucose, Perceived Stress Scale (PSS), Pharmaceutical Benefits Scheme (PBS), increased glucose level, saliva, serum

## Abstract

Oral fluids provide a readily available and non-invasive medium for the diagnosis of a wide range of diseases and clinical situations. Diabetes mellitus is a common chronic metabolic disorder that affects millions of people. Our objective was to compare the salivary and serum glucose levels in patients with diabetes mellitus and healthy individuals. Two ml of unstimulated whole saliva was collected by the spitting method. Also, 2 ml of the patient’s intravenous blood was obtained from the forearm’s median cephalic vein. Both the samples were centrifuged at 2000 rpm for 2-3 minutes. Ten μl of both saliva and serum were taken out and added to glucose reagent. These were kept in a temperature-controlled water bath at 37°C for 10 minutes. The color change was noted, and the optical density was measured in a semi-auto analyzer.

The presence of glucose was detected in both groups; however, the levels were raised in people with diabetes compared to healthy individuals. The present study indicated a substantial increase in salivary and serum glucose levels in diabetic patients compared to healthy controls. The concentration of glucose in saliva increases with the increase in serum glucose concentration.

## Introduction

Diabetes Mellitus (DM) is probably one of the oldest diseases known to man. It was first reported in Egyptian manuscripts about 3000 years ago [1]. It is an endocrine and/or metabolic disorder with a growing prevalence rate and worldwide incidence [2]. The prevalence in 2000 was estimated to be 2.8% and is expected to increase to 4.4% by 2030 [3].

DM is represented by chronically increased glucose level assigned to the autoimmune destruction of pancreatic beta (β)-cells of the pancreas with insulin inadequacy to abnormalities that result in insulin resistance [4]. The deficient insulin action on target tissues, mainly striated muscle fibers, adipose tissue, and liver, forms the basis of carbohydrate, fat, and protein metabolism disturbances [5].

The specific features include a triad of polyuresis, excessive thirst and polyphagia, which may be accompanied by weight reduction and hazy vision [6]. Acutely increased glucose level causes increased urine excretion (polyuresis) and, as a result, excessive thirst, water ingestion. These presenting symptoms of DM are also termed “osmotic-symptoms” [7]. There might be impaired growth and susceptibility to certain infections. The debilitating effects of DM include various organ collapses, progressive metabolic complications such as retinopathy, nephropathy, and/or neuropathy [2]. Uncontrolled diabetes may lead to stupor, coma, and, if not treated, death (due to ketoacidosis or, rarely, hyperosmolar hyperglycemic nonketotic coma) [8].

DM can be broadly classified into four types: type 1 and 2, pregnancy-related diabetes, and other specific types [9]. 85-90% of the patients have type 2 diabetes, whereas only 5 to 10% have type 1 DM. Type 1 DM, also known as juvenile diabetes, mainly affects young individuals and is characterized by autoimmune destruction of pancreatic beta cells, leading to absolute insulin inadequacy. Type 2 DM is considered an adult disorder as it usually develops in patients older than 40 years, and it frequently is associated with overweight or obese phenotypes and is characterized by constitutional insulin resistance with relative insulin deficiency [10].

There is a wide spectrum of oral complications associated with DM linked with the degree of glycemic control. These may include gingivitis and periodontal disease, xerostomia, increased predisposition to bacterial, viral and fungal infections, impaired ability to wear dental prostheses, dysgeusia, and burning mouth syndrome [11].

Saliva is one of the most important exocrine secretions and plays an important role in the management of oral and digestive conditions. It not only contributes to speech, mastication and swallowing but also a useful systemic sampling tool for medical diagnosis and research. It can be collected easily by non-invasive methods [12].

Early diagnosis and prompt treatment are key to preventing complications, including peripheral neuropathy with risk of foot ulcers, amputation, and Charcot arthropathy, and autonomic neuropathy, causing gastrointestinal, genitourinary, and cardiovascular symptoms and sexual dysfunction, nephropathy leading to renal impair, and retinopathy which can cause blindness. Research says that the needle used to withdraw blood causes discomfort and can discourage patients from properly monitoring their blood sugar levels. The current study wants to find out an approach that can be used to diagnose and monitor diabetes. Saliva is one of the most abundant secretions in the human body and can be very easily obtained [13].

The risk of complications associated with diabetes is largely determined by the quartet of age, obesity, family history, and ethnicity. Type 2 diabetes targets the rich people of developing countries and the poor people of developed countries. Prevalence estimates vary according to access to diagnostic facilities, the diagnostic cut-offs used at the time of the survey, the means of ascertainment, the nature and age-structure of the population under consideration, the ability to distinguish between type 1 and type 2 diabetes, and the longevity of those affected. Despite all these reasons for variation, recent estimates are consistent in showing a rising prevalence of diabetes around the world [14].

The National Diabetes Data Group and the World Health Organization (WHO) have issued diagnostic criteria for DM:

• Symptoms of diabetes plus occasional plasma glucose concentration of ≥ 200 mg/dl (11.1 mmol/l). The classic symptoms of diabetes include polyuresis, excessive thirst, and unexplained weight reduction.
• Fasting Plasma Glucose ≥ 126 mg/dl (7.0 mmol/l). Fasting is defined as no caloric intake for at least 8 hours.
• Two-hour postprandial glucose ≥ 200 mg/dl (11.1 mmol/l) during an Oral Glucose Tolerance Test. The test should be performed as described by the WHO, using a glucose load containing the equivalent of 75g of anhydrous glucose dissolved in water [15].

Glycosylated (or glycated) hemoglobin (HbA1c, A1C) is a type of hemoglobin utilized mostly to recognize the normal plasma glucose concentration over delayed timeframes. Sacks et al. dissected and looked into the different lab strategies used to analyze and screen diabetes. They assessed that glycated hemoglobin is a clinically valuable list of mean glycemia values during the previous 120 days, the average life expectancy of erythrocytes. There is a wide range of strategies extending from low-throughput examine research center segment frameworks and manually scaled-down segment techniques to high-throughput robotized frameworks devoted to GHb determinations. They have discovered that the aftereffects of different test standards show astounding connection, and no strategy is unmistakably better than the rest [16].

Saliva is a complex fluid produced by the salivary glands, an essential function of which is to maintain the well-being of the mouth. It has become a helpful fundamental sampling tool for clinical conclusions and research as it can be gathered effectively and non-invasive. The presence of glucose in the salivary secretion is a settled actuality, although it was accepted beforehand that salivation does not contain glucose as a typical constituent, and the mean values did not depend on the gender [17, 18].

As indicated by past investigations, the connection between salivary glucose and serum glucose was reliable in both the diabetics and controls. Henceforth, they presumed that salivary glucose gives off an impression of being a solid pointer of serum glucose concentrations, especially in diabetic patients [19].

The salivary glucose concentration was higher in diabetic patients than control subjects, regardless of whether the saliva was stimulated or not [20].

The point of this research was to discover the practicality of saliva in diabetes diagnosis and to determine its demonstrative and prognostic value.

We have directed this investigation to assess the analytic and prognostic significance and compare the salivary and serum glucose level in patients with DM and correlate the data with that of healthy subjects.

## Material and Methods

The investigation was done at the Department of Oral Pathology and Microbiology, Patna Dental College and Hospital, Patna (Bihar) for half a year from January 2019 to July 2019. Two hundred subjects were chosen from the out-patient department and were split into four groups: Group I - 50 subjects with type 1 DM; Group 2 - 50 people with type 2 DM; Group 3, 50 subjects with recently diagnosed DM and Group 4 with 50 healthy subjects. All the procedures that were performed in this research were as per the ethics of the University Foundation. Informed consent was obtained from all patients and volunteers.

Regarding sample collection, 2 ml of unstimulated saliva were gathered by the spitting technique. Also, 2 ml of the patients’ intravenous blood were drawn from the middle cephalic vein of the lower arm. The examples were then moved to a fluoride tube. Both samples were centrifuged at 2000 rpm for 2-3 minutes. The glucose estimation unit was first reconstituted by dissolving the working reagent in a powder structure in the glucose diluents and put away in a dry golden shaded bottle. The reconstituted reagent is steady for in any event 90 days at 2 - 8°C. Both the examples were centrifuged at 2000 rpm for 2-3 minutes. The supernatant was utilized to gauge the glucose. 1000 μl of the working reagent was included in 3 separate sterile test tubes, and 10 μl of deionized water was added to the primary cylinder (Blank). Then, 10 μl of the standard glucose arrangement containing 100 mg/dl of glucose was added to the subsequent cylinder (Standard). 10μl of salivation/serum test was added to the third cylinder (Test). All the substances in the three cylinders were completely blended and incubated for 15 minutes at 37°C. Absorbance was read by a semi-autoanalyzer (Accurex). Blank and Standard absorbance were then estimated. The analyzer was pre-modified to give the estimation of standard glucose, which was utilized as the baseline to assess the glucose substance of the Test. When test absorbance was estimated, the analyzer consequently gave the test glucose content.

## Results

The study group comprised of 150 diabetic subjects and 50 non-diabetic (control) subjects. Among the study group, type 1 diabetes - 15 (30%) subjects were below 45 years of age while 35 (70%) were ≥ 45 years of age; type II diabetes- 24 (48%) subjects were below 45 years of age, and 26 (52%) were ≥ 45 years of age. Among newly diagnosed diabetic patients, 23 (46%) were below 45 years of age, and 27 (54%) were ≥ 45 years old. Among the control group, 43 (86%) subjects were below 45 years of age and 7 (14%) were ≥ 45 years of age (Table 1). Among type I diabetic patients, 32 (64%) were male, and 18 (36%) female. Among type II diabetic patients, 30 (60%) were male and 20 (40%) female. Among newly diagnosed diabetic patients, 54% were male, and 46% female. Among the control group, 29 (58%) were male, and (42%) female (Table 2).

**Table 1:** Age distribution of patients in study groups.

**Study Groups**	**Age**	
	**<45 years**	**≥ 45 years**
**Type-I Diabetes**	15 (30%)	35 (70%)
**Type-II Diabetes**	24 (48%)	26 (52%)
**Newly Diagnosed Diabetes**	23 (46%)	27 (54%)
**Control**	43 (86%)	7 (14%)

**Table 2:** Gender distribution of patients in the study group.

**Study Groups**	**Male**	**Female**
**Type-I Diabetes**	32 (64%)	18 (36%)
**Type-II Diabetes**	30 (60%)	20 (40%)
**Newly Diagnosed Diabetes**	27 (54%)	23 (46%)
**Control**	29 (58%)	21 (42%)

Among the 50 type I diabetic subjects, 12 had a PBS value between 101 and 200 mg/dl with a mean value of 10.833 ±2 .0816 PSS level, 30 had a PBS value between 201 and 300 mg/dl with a mean value of 1.167 ± 1.555 PSS level, and 8 had a PBS value > 300 mg/dl with a mean value of 16.3750 ± 2.352 PSS level. Among the type II diabetic patients, all 50 had a PSS level of 13.12 ± 2.352. Among the 50 newly diagnosed diabetic patients, 44 had a PBS value between 201 and 300 mg/dl with a mean value of 12.8864 ± 1.31566 PSS level, and 6 had a PBS value> 300 mg/dl with a mean value of 19.0000±2.44022 PSS level. In newly diagnosed diabetic patients, all 50 subjects had 13.6200 ± 2.44022 PSS level (Table 3).

**Table 3:** Comparison of descriptive statistics between PBS and PSS amongst various groups.

**Study groups**	**PBS** **(mean±SD)**	**PSS** **(mean±SD)**
**Control**	131.86 ± 15.037	6.36 ± 0.693
**Newly diagnosed Diabetes**	266.16 ± 47.036	13.62 ± 2.44
**Diabetes – I Diabetes**	245.02 ± 49.835	13.12 ± 2.353
**Diabetes – II Diabetes**	233.08 ± 6.491	11.46 ± 3.22

Among type II diabetic patients, out of the 50 subjects, 24 had a PBS value between 101-200 mg/dl with 9.2083 ± 0.83 PSS level, 20 subjects had a PBS value between 201-300 mg/dl with a mean value of 12.0 ± 1.654 PSS level and 6 subjects had a PBS value >300 mg/dl with a mean value of 18.6667 ± 0.816 PSS level. Among type II diabetic patients, all 50 subjects had a PSS level of 11.4600 ± 3.228.

In the control group, a statistically substantial difference was found in average PBS values between subjects aged <45 years and >45 years while there was no significant difference found in the average PSS value for the same patients. In the newly diagnosed group, there was a statistically substantial difference found in average PBS values between subjects aged less or more than 45 years, while no significant difference was found regarding the PSS value. In the diabetes type I group, there was a statistically substantial difference found between subjects aged <45 years and >45 years in PBS while no significant difference was found regarding PSS. In the type II diabetes group, there was a statistically substantial difference found between subjects aged <45 years and >45 years regarding PBS, while no significant difference was found concerning PSS.

There was a substantial difference in the average values of PBS and PSS between male and female subjects in all study groups.

## Discussion

DM is a group of metabolic diseases characterized by chronically increased glucose levels resulting from defects in insulin secretion and/or insulin action. Insulin deficiency and/or insulin resistance of target tissues, mainly striated muscle fibers, adipose tissue, and to a lesser extent, liver, at the level of insulin receptors, signal transduction systems, and/or effector enzymes or genes are responsible for these metabolic abnormalities [21]. As implied by the American Diabetes Association (ADA) in 1997, it can broadly be classified as type 1, type 2, other types, and gestational DM. The constant increased glucose level is related to long-term damage, dysfunction, and failure of various organs, particularly the eyes, kidneys, nerves, heart, and veins.

A triad of polyuria, polydipsia, and polyphagia is distinguished in the clinical picture of DM, along with weight loss and impaired vision. These symptoms may also coexist with impaired growth and a tendency to specific infections. In acute conditions, uncontrolled diabetes can lead to hyperglycemia with ketoacidosis or hyperglycemic hyperosmolar nonketotic coma. Long-term complications include retinopathy that can lead to vision loss, nephropathy, which promotes renal failure, peripheral neuropathy with a high risk of foot ulcers and amputations, Charcot foot, and autonomic neuropathy causing gastrointestinal, genitourinary, and cardiovascular symptoms and impaired sexual function [22].

Recently, the International Diabetes Federation denoted that DM afflicts around 8.3% of adults (382 million individuals). This figure may rise further by 592 million in not more than 25 years. With 175 million undiagnosed cases of DM at present, an enormous crowd is progressing unprepared in the direction of complications [23].

Blood is taken as an analytical body fluid for diagnosis, with a wide variety of available devices in the market to determine glucose levels. There has been a need to establish a non-invasive procedure to evaluate the glucose level without pricking. Saliva offers an imperative role in the homeostasis as it stabilizes the ecosystem of the oral cavity, and therefore, it serves as a brilliant marker for glucose level estimation. Glucose is a constituent that can cross the salivary gland epithelium in proportion to its blood concentration. Of all salivary parameters, salivary glucose appears to be most closely related to the oral environment in patients with diabetes [24].

In the current study, the glucose concentration in unstimulated whole saliva was analyzed. Stimulated whole saliva is inappropriate for the diagnostic goal of the study since the foreign substances used as salivary stimulants tend to alter the pH and generally stimulate the serous salivary secretion, resulting in a dilution in the concentration of molecules of interest.

The present study comprised of 150 diabetic subjects and 50 non-diabetic (control) subjects. This investigation aimed to decide whether any considerable connection existed between the postprandial serum and salivary glucose levels. The postprandial salivary glucose levels were assessed in diabetic patients and healthy controls.

A measurably significant correlation was found among salivary and serum glucose levels in patients with diabetes and controls (P < 0.01). Our outcomes were in concordance with the research led by Carlson et al., who revealed the presence of glucose in diabetic patients’ saliva alongside an increase in salivary glucose levels in DM patients in contrast with healthy controls [25]. However, our results were in opposition to the discoveries announced by Forbat et al. They presumed that salivary glucose levels did not reflect blood glucose levels [26]. Carda et al. concluded that the salivary glucose levels of most diabetic patients were in the normal range [27]. On the other side, Thorstensson et al. reported an increase in salivary glucose levels in DM patients compared to non-diabetics [28].

A statistically significant difference was found regarding the normal values of PBS between the subjects of the study and control groups, while there was no significant difference found in average PSS values.

Pearson’s correlation coefficient was utilized to assess the relationship between salivary glucose and serum glucose in diabetics and controls. A significant correlation existed among salivary and serum glucose in diabetic patients and controls. Hence, it seems that salivary glucose can indicate serum glucose concentration in diabetic patients.

Reuterving et al. led a study on diabetic patients and performed salivary examinations. Patients with DM experienced salivary examinations on two events during various metabolic control situations. A positive relationship between glucose levels in saliva and blood was noticed in stimulated parotid saliva. There were no significant contrasts regarding pH, buffering limit, the total amount of protein, amylase, lysozyme, peroxidase, or electrolytes (Na+, K+, Ca+2, PO4-2 and Mg+2) in the saliva. Salivary glucose levels were lower during the time of better metablic control [28].

Belazi et al. conducted a study to inspect the flow rate and composition of unstimulated whole saliva and serum in children with recently analyzed insulin-dependent DM and contrasted those with healthy controls. Their discoveries indicated that the glucose levels in the unstimulated whole saliva, as well as in the serum of insulin-dependent DM group were higher compared to healthy subjects. They reasoned that the expanded permeability of the basement membrane in insulin-dependent DM might lead to an enhanced leakage of serum-derived components into the whole saliva via gingival crevices. Consequently, a considerable increase in salivary glucose levels in patients with insulin-dependent DM could be manifested [29].

In another study, Jurysta et al. evaluated the salivary glucose concentration and excretion in unstimulated and mechanically stimulated saliva in both healthy and diabetic subjects. They discovered that the salivary glucose concentration and excretion were a lot higher in diabetic patients compared to control subjects, regardless of testing stimulated or unstimulated saliva. In diabetic patients, when compared with control subjects, the magnitude of the increase in saliva glucose concentration was practically identical to that of blood glucose concentration [30].

Sreedevi et al. found an exceptionally significant correlation between salivary glucose and serum glucose before the treatment and diabetes control in their study of comparison of serum glucose and salivary glucose in diabetic patients. The connection between salivary glucose and serum glucose was also significant in controls. The degree of salivary glucose did not vary with age and sex. The relationship between salivary glucose and serum glucose was solid in both people with diabetes and controls. Subsequently, they presumed that salivary glucose seems, by all accounts, to be a solid indicator of serum glucose levels, especially in diabetic patients [19]. There was no considerable distinction in salivary glucose when differentiated among various age groups, and a slight male predilection was noticed. Salivary glucose concentration was directly proportional to that of serum glucose. These discoveries were consistent with the results reported by Sreedevi et al. [19].

In their study, Shehla Amer et al. assessed salivary and blood glucose levels in non-diabetics and patients with DM. Glucose was found only in the saliva of patients with DM, while the salivary samples of non-diabetic subjects of the same age did not show the presence of glucose. A significant relationship was found between the glucose level from saliva and blood in diabetic patients. As indicated by them, this finding proposes that saliva can be utilized to reflect and observe blood glucose levels in patients with DM [31].

## Conclusion

The current investigation reveals that salivary glucose has increased levels in the saliva of patients with diabetes. A significant relationship was found among salivary and serum glucose levels in type I, type II diabetes patients and healthy subjects. Saliva has an essential role in the homeostasis of the oral cavity because it stabilizes the ecosystem of the oral cavity, and therefore, it serves as an excellent marker for early detection of many conditions. Salivary glucose is most closely related to the oral environment in patients with diabetes. Glucose, a small molecule, can easily diffuse through semi-permeable membranes, resulting in an increase in the salivary glucose levels, which is attributed to the reduction of homeostasis and greater susceptibility to oral diseases. Since the collection of saliva samples is safe and comfortable, it offers benefits over blood drawing in children, elderly, critically ill, and debilitated patients and can be used as non-invasive biomarker for screening, diagnosing, and monitoring of diabetes.

## Conflict of Interest

The authors declare that there is no conflict of interest.
